# Establishment and identification of cardiomyocyte arhGEF18 gene conditional knockout mice

**DOI:** 10.1002/pdi3.20

**Published:** 2023-08-14

**Authors:** Kun Wan, Wenjing Yuan, Tiewei Lv

**Affiliations:** ^1^ Department of Cardiology Children's Hospital of Chongqing Medical University National Clinical Research Center for Child Health and Disorders Key Laboratory of Child Development and Disorders of Ministry of Education Chongqing Key Laboratory of Pediatrics Chongqing China; ^2^ Sichuan Vocational College of Health and Rehabilitation Zigong Sichuan China

**Keywords:** arhGEF18, conditional knockout, Cre/Loxp, left ventricular noncompaction

## Abstract

Left ventricular noncompaction (LVNC) is a heterogeneous disorder with unclear genetic causes. The arhGEF18 gene is a guanine nucleotide exchange factor related to the Rho pathway and a possible predisposing gene for LVNC. In this study, a mouse model of arhGEF18 gene conditional knockout (cKO) in cardiomyocytes was established using the Cre‐LoxP system to provide an animal model for the genetic study of LVNC. ArhGEF18 cKO mice were obtained by crossing arhGEF18^f/+^/myh6‐Cre^+^ and arhGEF18^f/f^ mice. The mRNA and protein expression levels of arhGEF18 in different tissues were verified. The myocardial cytoskeleton and polar protein expression, the survival rate, cardiac function, and cardiac histology of model mice were determined. ArhGEF18 cKO mice were successfully established. ArhGEF18 was confirmed to be specifically knocked out in the myocardial tissue of arhGEF18^f/f^/myh6‐Cre^+^ mice at the mRNA and protein levels, and the knockout rate was approximately 75%. The relative expression levels of cytoskeleton and cell polarity proteins such as α/β‐tubulin, Scribble, Crb2, and PAR3 in the cKO group were significantly lower than those in the control group (*p* < 0.05). During monitoring, the survival rate, cardiac systolic function, and myocardial structure of arhGEF18^f/f^/myh6‐Cre^+^ mice were not significantly different from those of control mice. A mouse model of cardiomyocyte arhGEF18 gene cKO was successfully established, and it was confirmed that knocking out arhGEF18 changed cytoskeleton and cell polar protein expression levels in the myocardium. However, there was no obvious LVNC phenotype in the constructed arhGEF18 cKO mice.

## Graphical abstract

A mouse model of cardiomyocyte arhGEF18 gene conditional knockout was successfully established. It was confirmed in model mice that knocking out the arhGEF18 gene can change the expression levels of cytoskeleton and cell polar proteins in the myocardium. However, there is no obvious phenotype of left ventricular noncompaction (LVNC) in the constructed arhGEF18cKO mice at present. The model mice will be given external pathological stimuli or load tests to further study the role and mechanism of arhGEF18 in relation to LVNC.

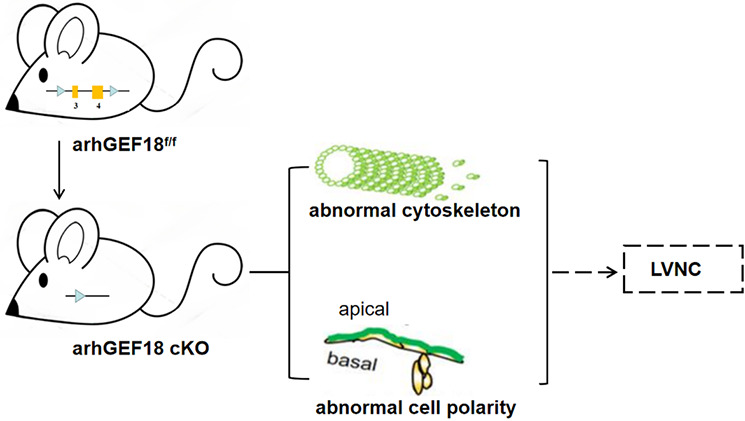

## 1 INTRODUCTION

Left ventricular noncompaction (LVNC) is a common cardiovascular disease in children. It is characterized by prominent and excessive trabeculation with deep recesses in the ventricle, especially the left ventricle. The main clinical manifestations are progressive heart failure, arrhythmia, thrombosis and embolic events, and even sudden death. There is no effective treatment at present.[^1^, ^2^] It is a serious cardiovascular disease that threatens children's health and increases the burden on families and society. LVNC is a recognized genetic cardiomyopathy that is affected by genetic factors, environmental factors, and their interaction. To date, genetic studies of LVNC based on genome‐wide or exome‐wide association analyses have identified dozens of disease‐related susceptibility genes, including those associated with sarcomeres (MYH7, ACTC, TNNT2, MYBPC3, TMP1, ZASP/LDB3, and TNNI3),[^3^, ^4^] ion channel‐related genes (SCN5A, HCN4, TAZ, and LMNA)[^5^, ^6^] and mitochondrion‐related genes (mtDNA, ND1, and Cytb),^[7]^ but these genes explain only 20%–30% of the disease; most of them are found in Western populations, and only a few have been reported in the Chinese Han population, which cannot fully explain the genetic basis of LVNC in Chinese people. More importantly, there is a lack of verification of pathogenicity from the perspective of phenotypic changes in animal models.

A typical LVNC family was discovered by our research team in the early stage. Whole‐exon sequencing was performed on the peripheral blood of the proband and his family (parents and sister). Meanwhile, transcriptome sequencing was performed on the induced pluripotent stem cell‐myocardial cell line derived from the proband, and combined analysis was performed to screen out the possible predisposing gene arhGEF18 (p114RhoGEF) for LVNC occurrence. We intend to verify the function and explore the mechanism of the arhGEF18 gene in LVNC occurrence at the molecular, cellular, and model animal levels. In this study, we used the Cre/Loxp system to construct and identify an arhGEF18 gene knockout mouse model, which will provide an animal model for subsequent genetic research on LVNC.

## 2 MATERIAL AND METHODS

### 2.1 Mice

ArhGEF18^f/+^ and Myh6‐Cre^+^ transgenic mice with a genetic background of C57BL/6J were purchased from Saiye Model Biology Research (Taicang) Co., Ltd. Wild‐type C57BL/6J mice were purchased from Spafford (Beijing) Biotechnology Co., Ltd. All mice were housed in a specific pathogen‐free room in the Animal Center of Children's Hospital of Chongqing Medical University. The temperature was controlled at 20–25°C, and the humidity was 50%–60%. The cages and bedding of mice were changed twice a week. A male to female ratio of 2:1 was used for breeding. The experimental procedures described in this study were approved by the Experimental Ethics Committee of Children's Hospital of Chongqing Medical University (license number: CHCMU‐IACUC2021 1028001).

### 2.2 Construction process of arhGEF18 cKO mice

**FIGURE 1:**
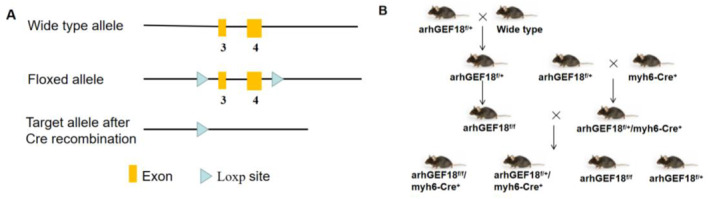
Construction strategy and breeding flow chart of arhGEF18 conditional knockout mice. (A) Construction strategy schematic diagram and (B): Breeding flow chart.

The arhGEF18^f/+^ mouse is a transgenic heterozygous mouse that contains loxP sites flanking exons 3 and 4 of the arhGEF18 gene. After mating with transgenic mice expressing Cre recombinase, the sequence between the two loxp sites in a specific tissue or cell can be excised, and the construction strategy is shown in Figure 1A. The arhGEF18^f/+^ mice were constructed by Saiye Company and then used for subsequent reproduction. A total of three generations of breeding were required to obtain arhGEF18 conditional knockout (cKO) mice: F1, arhGEF18^f/+^ mice were crossbred with wild‐type C57BL/6J mice to obtain many arhGEF18^f/+^ mice; F2, some of the arhGEF18^f/+^ mice obtained were bred with myh6‐Cre^+^ mice to obtain arhGEF18^f/+^/myh6‐Cre^+^ heterozygous mice, and the other arhGEF18^f/+^ mice were self‐crossed to obtain arhGEF18^f/f^ mice; and F3, ArhGEF18^f/+^/myh6‐Cre^+^ mice and arhGEF18^f/f^ mice were bred to obtain target mice. According to Mendelian laws of inheritance, the four genotypes of their offspring were arhGEF18^f/f^/myh6‐Cre^+^, ArhGEF18^f/+^/myh6‐Cre^+^, ArhGEF18^f/f^, and arhGEF18^f/+^ (the last two genotypes were both wild type), each accounting for 1/4 of the offspring. The breeding flow chart is shown in Figure 1B.

### 2.3 Genotyping

Genotyping is an essential step in the breeding of mice. Genomic DNA was extracted from mouse tails by using the mouse tail direct PCR rapid genotyping kit (Bimake). The arhGEF18‐loxp genome and myh6‐Cre genome were genotyped separately according to the kit instructions. Primers for the genes were synthesized by Life Technologies Corporation (Shanghai, China), and the sequences of the primers were as follows: arhGEF18‐loxp (F), 5′‐GCTTCAGACTCACATAGCACTGAG‐3′; arhGEF18‐loxp (R), 5′‐TAGCTAGGTAGCACAAGCCTCA‐3′; Myh6‐Cre (F), 5′‐ATGACAGACAGATCCCTCCTATCTCC‐3′; and Myh6‐Cre (R),5′‐CTCATCACT CGTTGCATCATCGAC‐3′.

### 2.4 Verification of the knockout effect by qPCR and western blot analysis

To verify the knockout effect, four 1‐month‐old arhGEF18^f/f^/myh6‐Cre^+^ mice were used as the experimental group and four littermate arhGEF18^f/f^ mice were used as the control group. The mice were euthanized by CO_2_ to obtain myocardial tissue (only the ventricle), lung tissue, liver tissue, and kidney tissue, which were stored at −80°C for subsequent quantitative real‐time PCR (qPCR) and Western blotting.

Total RNA was extracted from mouse myocardial tissue (30 mg from each mouse) using a total RNA extraction kit (BioFlux), and the concentration was determined by a protein and nucleic acid quantitative analyzer. cDNA was synthesized from 500 ng of total RNA using a reverse transcriptase kit (Accurate Biology). qPCR was performed using SYBR Green I (Bimake). Relative mRNA expression was determined using the 2^−ΔΔCt^ method. To verify whether the cKO mice had knockout of arhGEF18 only in myocardial tissue, other tissues were also used for comparison. Liver, lung, and kidney tissues were used for comparison, and the protocol was the same as before. The sequences of the primers were as follows: arhGEF18 (F), 5′‐AAGTTGAAGCAGACGGCAGA‐3′; arhGEF18 (R), 5′‐TTA GTGTGCGCACGTGATGA‐3′; GAPDH (F), 5′‐TGCCCAGAACATCATCCCT‐3′; and GAPDH (R), 5′‐GGTCCTCAGTGTAGCCCAAG‐3′.

Total proteins were extracted from ventricle, liver, lung, and kidney tissues of mice using RIPA Lysis Buffer (Beyotime) as per the manufacturer's instructions, and protein concentrations were determined by a BCA protein assay kit (Beyotime). Sample proteins were separated on an SDS‒PAGE gel and transferred into a 0.45‐μm PVDF membrane. After transferring, the membrane was blocked in 5% free‐fat milk in TBST for 1 h and then incubated with primary antibodies overnight at 4°C. Then, the membranes were washed with TBST three times, followed by incubation with a secondary antibody. Finally, the protein bands were visualized using chemiluminescence, and band intensity was analyzed and measured with Image Lab. The following antibodies were used: anti‐arhGEF18 (Invitrogen, GT1972, USA, 1:1000) and anti‐GAPDH (ZENBIO, 200306‐7E4, China, 1:2000).

### 2.5 Quantification of myocardial skeletal protein and polarity protein expression levels in mice

Ventricular myocardial tissues from four 1‐month‐old arhGEF18^f/f^/myh6‐Cre^+^ mice and four arhGEF18^f/f^ mice were taken to detect the expression levels of cytoskeletal proteins (α‐actinin, α/β‐tubulin and Vinculin) and polar proteins (Scribble, Crb2 and PAR3) in myocardial tissues by western blotting. The methods were the same as described before. The following antibodies were used: anti‐α‐actinin (Cell signaling, 6487, USA, 1:1000), anti‐α/β‐tubulin (Cell signaling, 2148, USA, 1:1000), anti‐vinculin (Sigma, V9131, 1:500), anti‐Scribble (proteintech, 27083‐1‐AP, China, 1:1000), anti‐Crb2(abcam, 156286, 1:1000), and anti‐PARD3 (proteintech, 11085‐1‐AP, 1:1000).

### 2.6 Survival analysis

Survival analysis was used to compare the survival rates of littermate wild‐type (arhGEF18^f/f^ and arhGEF18^f/+^), arhGEF18^f/+^/myh6‐Cre^+^, and arhGEF18^f/f^/myh6‐Cre^+^ mice at different time points. The numbers of the three kinds of mice were 23, 14, and 15, respectively.

### 2.7 Echocardiographic monitoring of cardiac function in mice

Cardiac function was monitored at 2 and 4 months of age in the arhGEF18^f/f^/myh6‐Cre^+^ mice versus arhGEF18^f/f^ mice. Mice were anaesthetized with isoflurane and placed in the supine position on the manipulator plate, and their extremities were fixed to the electrode plate slab. The heart rate of the mice was maintained between 460 and 510 beats/min, and a Vevo small animal ultrasound probe with a frequency of 30 MHz was selected to first obtain a long‐axis view of the parasternal left ventricle. Then, the probe was turned 90° clockwise to obtain a short‐axis view of the papillary muscle to perform B‐ and M‐mode ultrasound. The mean of the measured values for 3 cardiac cycles was taken.

### 2.8 HE staining

Heart tissues were removed from 3 mice each in the two groups aged 1 and 4 months, cut longitudinally, and placed into 4% paraformaldehyde for more than 24 h of full soaking and fixation. After gradient alcohol dehydration, paraffin‐embedded sectioning, baking, dewaxing, hematoxylin eosin (HE) staining, and sealing, the slices were placed under a microscope for observation, and images were captured with a full‐slide scanner.

### 2.9 Transmission electron microscopy

The left ventricular tissues of 1‐month‐old arhGEF18^f/f^/myh6‐Cre^+^ mice and arhGEF18^f/f^ mice were taken for transmission electron microscopy (TEM) examination. The tissues were cut into long strips approximately 1 cubic millimeter in size with a sharp blade in the fixative solution and then gently transferred into a vial containing the fixative solution with forceps, stored at 4°C, and sent to the electron microscopy room of Chengdu Lile Medical Experiment Center for examination after adequate fixation.

### 2.10 Statistical analysis

Statistical analysis was performed using GraphPad Prism 8.3. Two‐sample *t* tests were performed for two‐group comparisons. The results are expressed as the means ± SDs. Differences with *p* < 0.05 were considered statistically significant compared with the respective controls.

## 3 RESULTS

### 3.1 Establishment and genotype identification of arhGEF18 cKO mice

**FIGURE 2:**
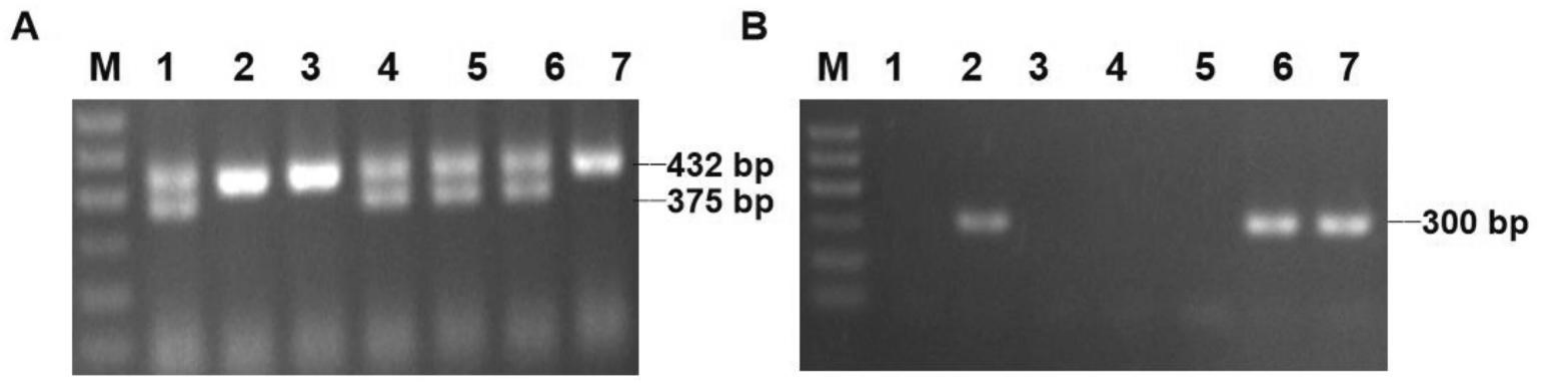
PCR analysis of genomic DNA in arhGEF18 conditional knockout mice. (A) ArhGEF18‐ loxp gene identification showed that 1, 4, 5, and 6 were heterozygous arhGEF18^f/+^, and 2, 3, and 7 were homozygous arhGEF18^f/f^. (B) Myh6‐Cre gene identification showed that 2, 6, and 7 were myh6‐Cre^+^, 1, 3, 4, and 5 were myh6‐Cre^‐^, and the M sample is the marker.

A total of 102 mice of the third generation were obtained, 49 female mice and 53 male mice, among which 27 were arhGEF18^f/f^/myh6‐Cre^+^ mice, 23 were arhGEF18^f/+^/myh6‐Cre^+^, 24 were arhGEF18^f/f^, and 28 were arhGEF18^f/+^. These numbers are basically consistent with Mendelian inheritance. The genotyping results for arhGEF18‐loxp were interpreted as follows: Only one band of 432 bp was arhGEF18^f/f^, only one band of 375 bp was the wild type, and both bands of 432 and 375 bp were arhGEF18^f/+^. The results of myh6‐Cre genotype identification were interpreted as follows: A single bright band of 300 bp was myh6‐Cre^+^, and no band was myh6‐Cre recombinase negative (Figure 2). In panels A and B of Figure 2, lanes 2 and 7 correspond to arhGEF18^f/f^/myh6‐Cre^+^ mice, that is, arhGEF cKO mice.

### 3.2 ArhGEF18 was specifically knocked out in mouse cardiomyocytes, as confirmed by qPCR and western blotting

**FIGURE 3:**
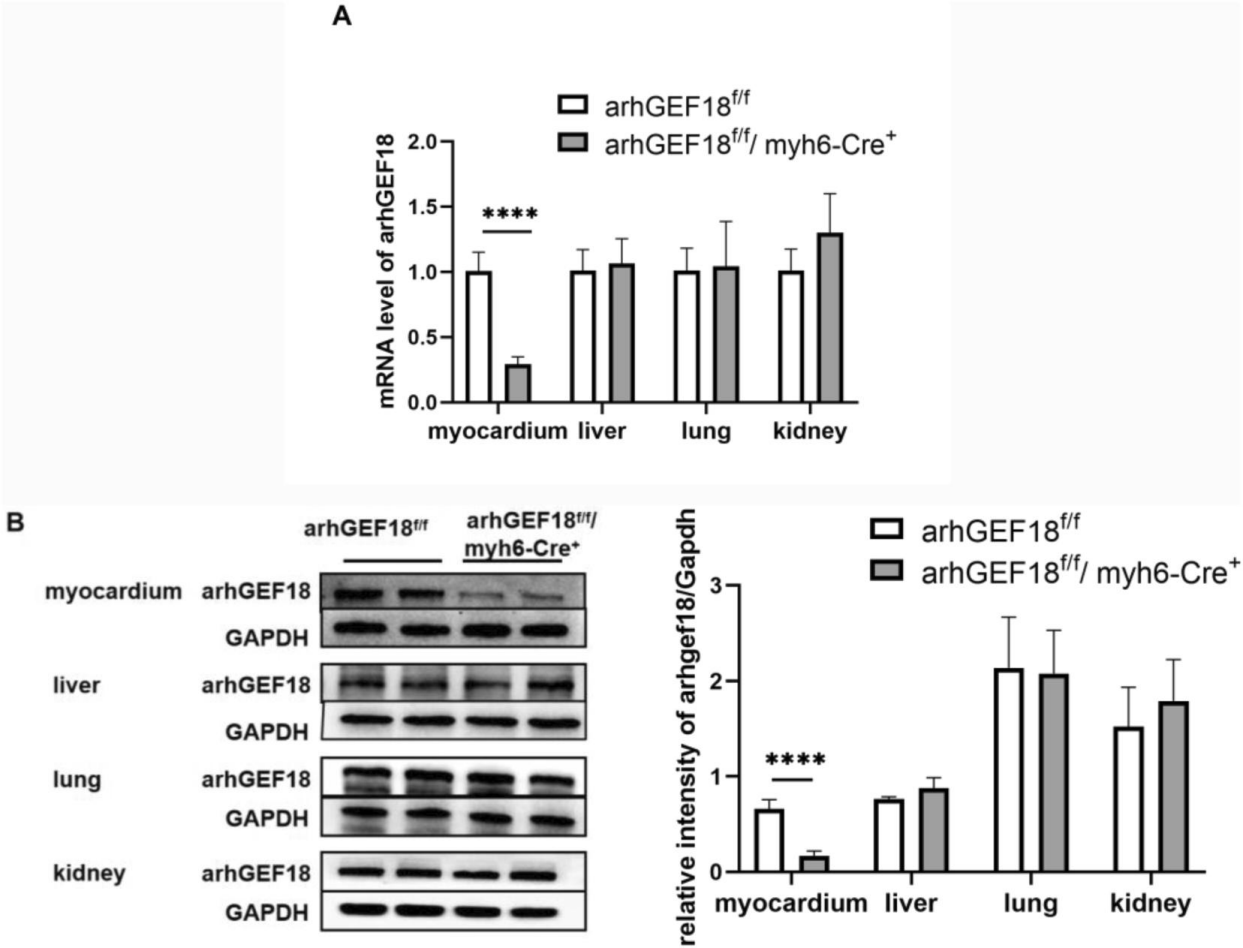
The expression of arhGEF18 mRNA and protein levels in the myocardium, liver, lung, and kidney of arhGEF18^f/f^ mice and arhGEF18^f/f^/myh6‐Cre^+^ mice. (A) The results of quantitative real‐time PCR showed that compared with that in arhGEF18^f/f^ mice, the mRNA level of arhGEF18 in the myocardium of arhGEF18^f/f^/myh6‐Cre^+^ mice was significantly decreased. (B) The western blot results showed that compared with that in arhGEF18^f/f^ mice, the protein expression of arhGEF18 in the myocardium of arhGEF18^f/f^/myh6‐Cre^+^ mice was significantly decreased. ****p < 0.0001. *N* = 4, Error bars show the mean (SD).

The results of qPCR showed that compared with that in the arhGEF18^f/f^ mice, the expression of arhGEF18 mRNA in the myocardial tissue of arhGEF18^f/f^/myh6‐Cre^+^ mice was significantly decreased by approximately 70% (1.007 ± 0.145 vs. 0.292 ± 0.057, *p* < 0.0001), while there was no significant difference in the expression of arhGEF18 mRNA in the liver, lung, or kidney tissues between the two groups (*p* > 0.05) (Figure 3A). Western blot analysis showed that compared with that in the arhGEF18^f/f^ mice, the protein expression of arhGEF18 in the myocardial tissue of the arhGEF18^f/f^/myh6‐Cre^+^ mice was also significantly decreased by approximately 75% (0.661 ± 0.094 vs. 0.168 ± 0.047, *p* < 0.0001), while the protein expression of arhGEF18 in the liver, lung and kidney tissues of the two groups showed no significant differences (*p* > 0.05) (Figure 3B). The above results confirmed that arhGEF18 was specifically knocked out in the cardiomyocytes of arhGEF18^f/f^/myh6‐Cre^+^ mice at the mRNA and protein levels.

### 3.3 The expression levels of cytoskeleton proteins and polar proteins in the mouse myocardium were altered

**FIGURE 4:**
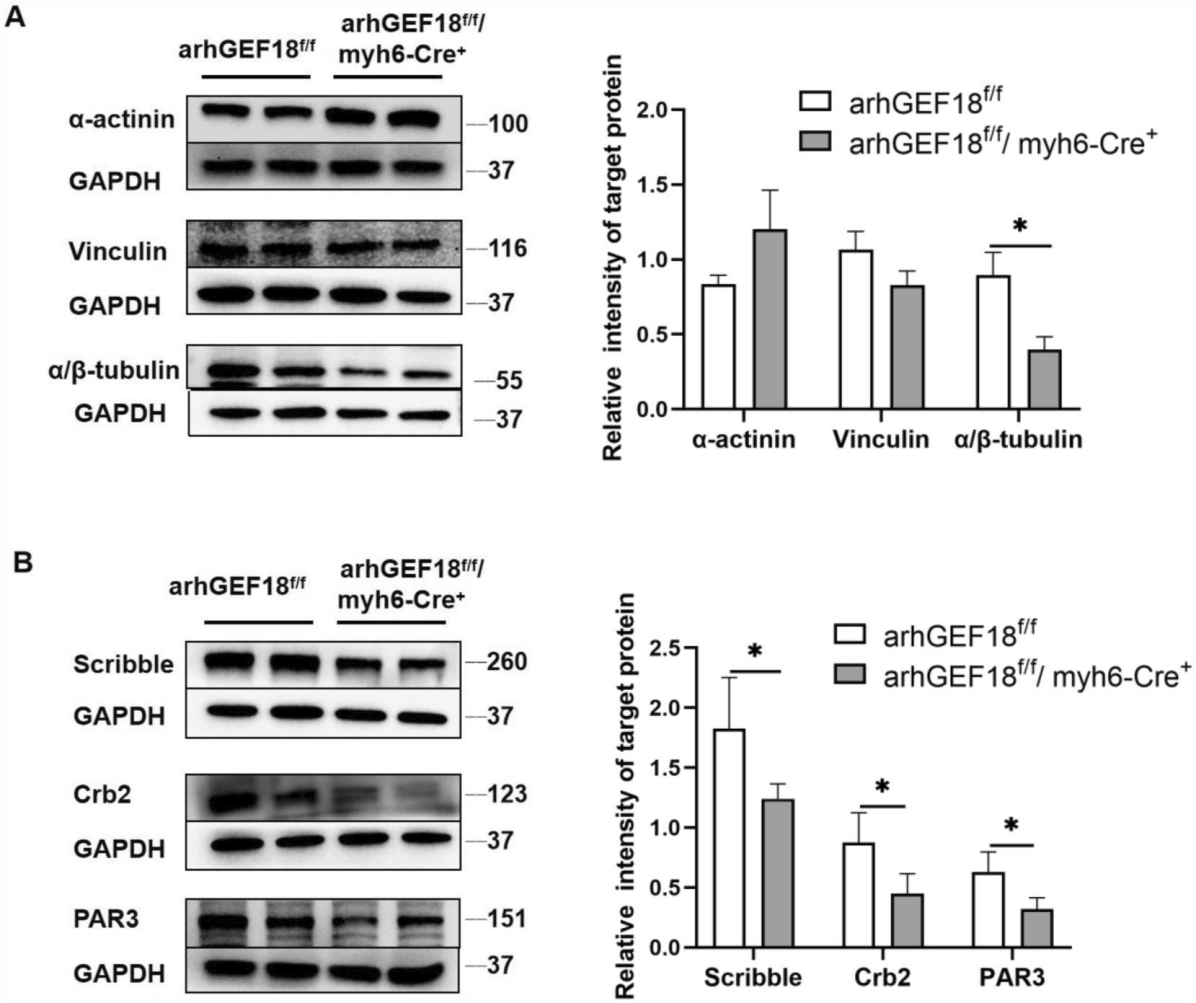
Western blot to detect the expression of cytoskeleton and polar proteins in the myocardium of mice, * indicates *p* < 0.05 versus the control group, *n* = 4 (A) expression level of cytoskeleton proteins and (B) expression level of cell polar proteins.

Western blotting was used to detect the expression of cytoskeleton‐related proteins (α‐actinin, α/β‐tubulin, and vinculin) and cell polarity‐related proteins (Scribble, Crb2, and Par3) in the mouse cardiac myocardium, and the results showed that the expression of α/β‐tubulin, Scribble, Crb2, and Par3 was more significantly reduced in the arhGEF18^f/f^/myh6‐Cre^+^ group than in the control group, and the results were statistically significant (*p* < 0.05), as shown in Figure 4.

### 3.4 Survival analysis

**FIGURE 5:**
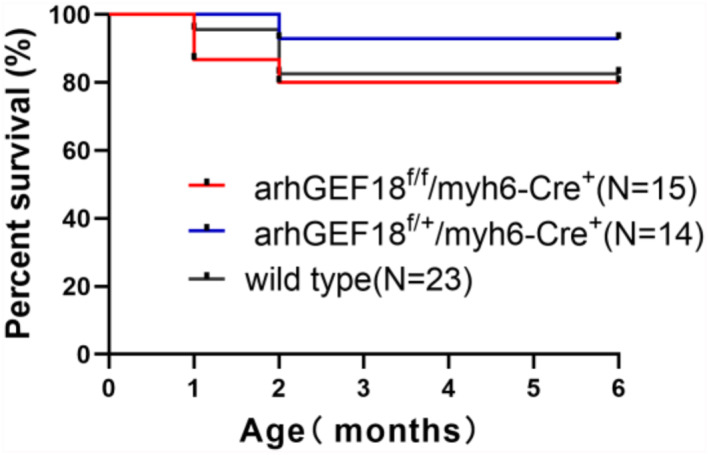
Survival curves of wild‐type, arhGEF18^f/+^/myh6‐Cre^+^, and arhGEF18^f/f^/myh6‐Cre^+^ mice.

Survival analysis was used to calculate statistics for the survival rates of the littermates of the wild type, arhGEF18^f/+^/myh6‐Cre^+^, and arhGEF18^f/f^/myh6‐Cre^+^ mice. A few mice of each type died in the first 2 months, and the cause of death may have been environmental factors or other reasons. At the age of 6 months, the survival rates of the three types of mice were all above 80%, with no significant differences (Figure 5).

### 3.5 Cardiac function in mice

**FIGURE 6:**
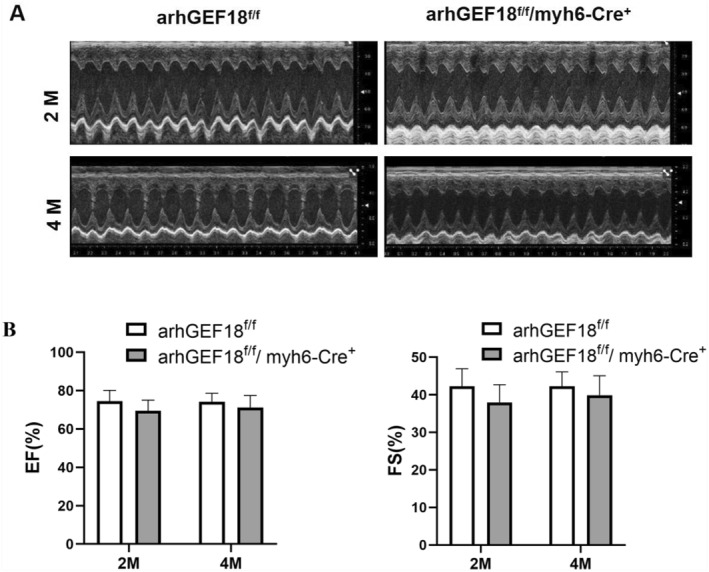
Echocardiogram and statistical diagram of cardiac systolic function of arhGEF18^f/f^ mice and arhGEF18^f/f^/myh6‐Cre^+^ mice. (A) Echocardiogram of the two groups and (B) statistical chart of EF and FS of cardiac systolic function.

Echocardiography of 2‐ and 4‐month‐old mice showed that there was no significant difference in the values of EF and FS of cardiac systolic function between the arhGEF18^f/f^/myh6‐Cre^+^ group and the arhGEF18^f/f^ group (*p* > 0.05) (Figure 6).

### 3.6 HE staining to observe ventricular structure

**FIGURE 7:**
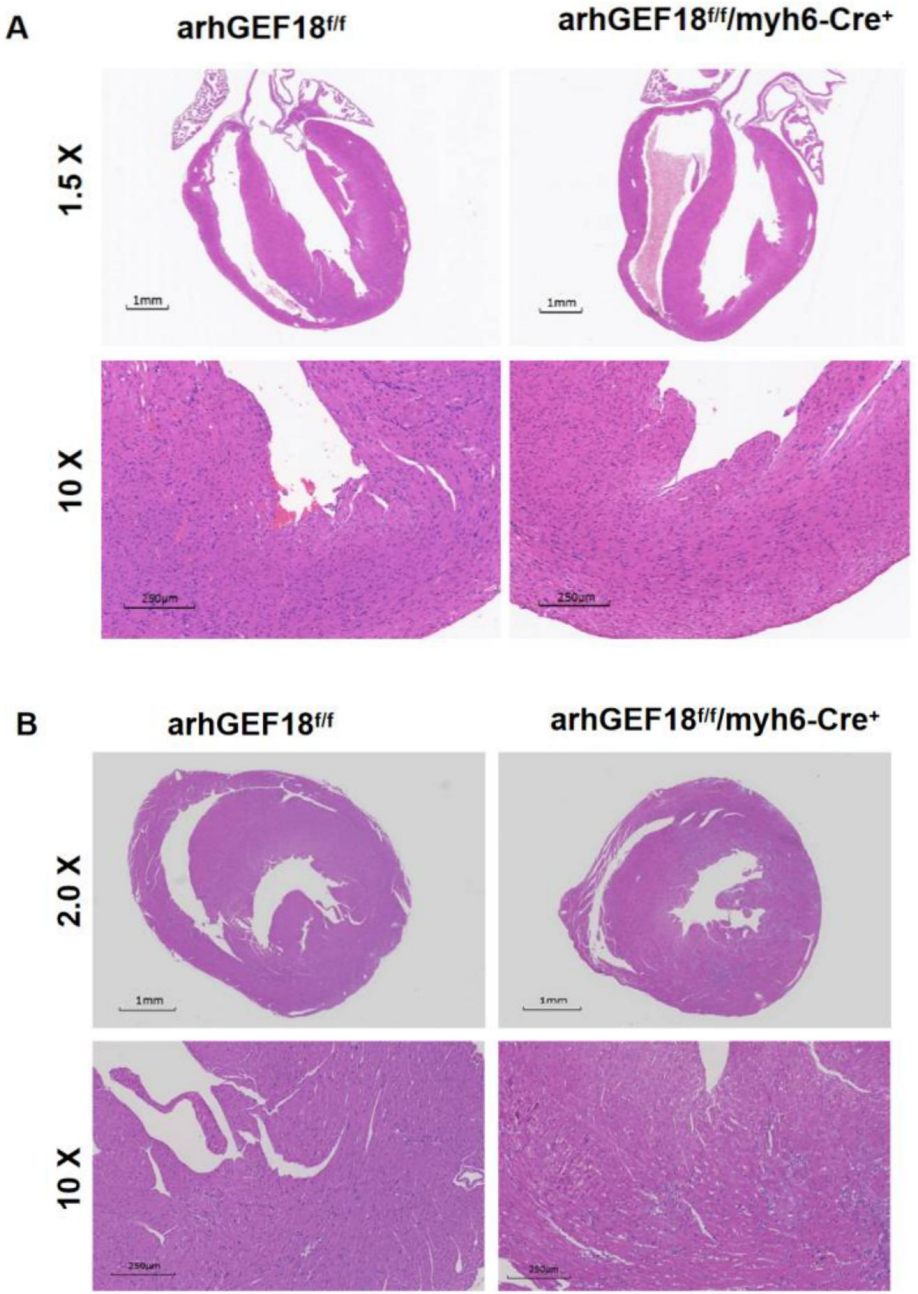
Hematoxylin eosin staining of paraffin sections of the heart tissue. (A) Four‐chamber section of the heart and (B) cross section of the heart near the apex.

After HE staining of the mouse heart tissue, the ventricular structure was observed under a microscope. The results showed that compared with the arhGEF18^f/f^ mice, there was no obvious thickening of the noncompacted myocardium and no significant abnormalities in the ventricular structure in the longitudinal section of the four chambers of the heart in the one‐month‐old arhGEF18^f/f^/myh6‐Cre^+^ mice (Figure 7A). At the age of 4 months, there was no obvious thickening of the noncompact myocardium in arhGEF18^f/f^/myh6‐Cre^+^ mice (Figure 7B).

### 3.7 Ultrastructure of left ventricle sarcomeres of mice

**FIGURE 8:**
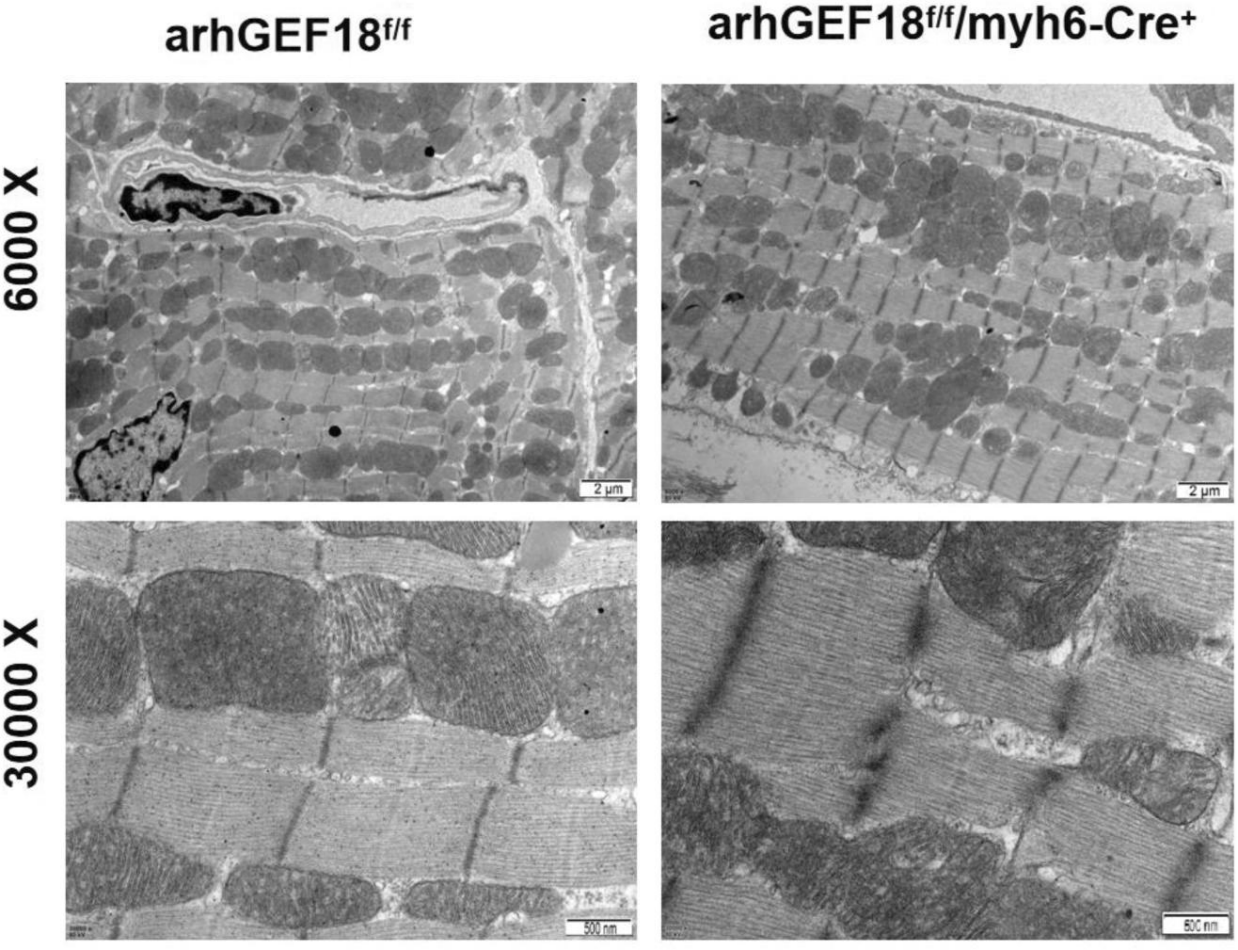
Ultrastructure of left ventricle sarcomeres of the two groups.

The TEM results for the left ventricular myocardium in arhGEF18^f/f^ mice and arhGEF18^f/f^/myh6‐Cre^+^ mice showed that the sarcomeres, Z line, and H band of myocardial cells in both groups were arranged neatly, and no obvious structural abnormality or disorder was found, as shown in Figure 8.

## 4 DISCUSSION

At present, LVNC is classified as a genetic disorder, but its pathogenesis remains unclear. Trabeculation is a meshwork of myocardial protrusions (trabeculae) that extend from the compact ventricular wall into the ventricular lumen in the developing heart. From late embryonic to perinatal stages, the trabecular myocardium undergoes compaction, resulting in the disappearance of trabeculae and thickening of the tightly packed ventricular wall, creating a relatively smooth endocardial surface. The delay or pause of this process is considered the pathological process that leads to the occurrence of LVNC.[^8^, ^9^] The formation of myocardial trabeculae and myocardial compaction is a complex evolutionary process, including not only the proliferation, differentiation, and maturation of cardiomyocytes but also cytoskeleton and cell polarity events, which play an important role in this process, so any mutation involved in this evolutionary process may represent the etiology of the disease. Elucidating the genetic mechanism of LVNC has become a research hotspot in recent years.

Many studies have reported that the cytoskeleton and cell polarity are involved in the noncompaction of the trabecular meshwork,[^10^, ^11^, ^12^] which is closely related to the occurrence of LVNC.[^13^, ^14^] The Rho pathway has been the most widely studied signal regulation pathway affecting the cytoskeleton and cell polarity in recent years.[^15^, ^16^, ^17^] Our team screened the predisposing gene arhGEF18 related to the Rho pathway in the early stage. The gene is a guanine nucleotide exchange factor (GEF), which contains a Db (DH) homologous domain and a closely linked pleckstrin (PH) homologous domain, in which the DH domain can catalyze the transformation of GDP into GTP and directly activate RhoGTPase, which is a GTP binding protein widely involved in cell function, plays a major role in cytoskeleton rearrangement, cell growth, and migration,[^18^, ^19^, ^20^] and has a high expression level in cardiac tissues. Reportedly, the arhGEF18 gene can regulate the polarity of neuroepithelial cells, promote retinal formation,^[21]^ and potentially affect the polarity of cells in the oral and facial regions, which is related to the occurrence of the cleft lip and palate.^[22]^ Mutation or deletion of this gene can also lead to an imbalance in Rho protein activation and hinder the arrangement of the cytoskeleton, cell proliferation, and the location of the polar complex.^[23]^ In addition, clinical studies on a large sample of Chinese people have shown that arhGEF18 gene mutation can increase susceptibility to nonidiopathic pulmonary hypertension complicated by coronary heart disease.^[24]^ However, there have been no reports about the relationship between the arhGEF18 gene and LVNC. According to the previous results obtained by our team, it is speculated that deletion of the arhGEF18 gene leads to an abnormal myocardial cytoskeleton and polarity by downregulating the Rho/ROCK pathway, which leads to the occurrence of LVNC. The clarity of this scientific hypothesis needs to be further verified at the molecular, cellular, and model animal levels. The construction of an animal model to identify the phenotypic changes after predisposing gene mutation is very important in functional verification.

Knockout mice are commonly used animal models for studying gene function. These mice include global knockout and cKO mice. Reportedly, arhGEF18 global knockout mice have obvious embryo lethality, and the main cause of death may be placental development defects, so it is impossible to obtain homozygous newborn mice with global knockout of the arhGEF18 gene.^[25]^ cKO is a site‐specific recombination technique using a system (the most common of which is the Cre‐LoxP system) on the basis of global knockout. This technique overcomes the defect of embryonic lethality caused by global knockout of some important genes and can more accurately reveal the specific role of target genes in specific cells or tissues. To date, extensive research has been carried out on this technique at home and abroad.^[26]^

In this study, an arhGEF18 myocardial cKO mouse model was established by using the Cre‐LoxP system. The birth ratio conformed to expectations under Mendelian inheritance, indicating that myocardial cKO of the arhGEF18 gene has no obvious embryonic lethality. The specific knockout of arhGEF18 in the cardiomyocytes of arhGEF cKO mice was confirmed at the mRNA and protein levels by qPCR and western blot. The knockout efficiency was approximately 75%. Approximately 25% of arhGEF18 remains due to the influence of other nonmyocardial cells in the heart, such as myocardial fibroblasts, vascular endothelial cells, and vascular smooth muscle cells. Therefore, a mouse model of arhGEF18 myocardial cKO was successfully constructed in this study. During monitoring, the survival rate, cardiac systolic function, and myocardial structure of arhGEF18^f/f^/myh6‐Cre^+^ mice were not significantly different from those of the control mice. The reasons are as follows: (1) Many reports show that after cKO of a gene, the morphology and function of the heart are not affected. However, once the stress factor or stress test is given, the corresponding phenotype of the deleted gene will appear, such as the TβR2 gene,^[27]^ Flt‐1 gene,^[28]^ and GRK2 gene.^[29]^ (2) This outcome may also be related to the Cre tool mouse. The Myh6‐Cre(ɑMyHC‐Cre) transgene directs the expression of Cre recombinase under the cardiac‐specific ɑ‐myosin‐heavy chain (Myh6) promoter, which affects over 90% of recombination in cardiomyocytes from adult and embryonic mice, starting from E8.5. Compared with some other myocardial cKO tool mice that can mediate recombination from E7.5 days, such as Nkx2.5‐Cre and cTnT‐Cre mice, the recombination starts slightly later; therefore, it has also been reported that there are different phenotypes after the same gene is knocked out by Myh6‐Cre mice and other cardiac cKO Cre tool mice with different promoters.[^9^, ^30^, ^31^]

This study also verified the expression of cytoskeleton proteins and polar proteins in the myocardial tissue of arhGEF18^f/f^/myh6‐Cre^+^ mice. It was confirmed that myocardial cKO of the arhGEF18 gene could affect the expression of cytoskeleton proteins and polar proteins, but there was no LNVC phenotype. This may be because the decrease in these cytoskeletons or polar proteins occurs later than the critical period of E12.5–E18.5 in the formation of mouse cardiac muscle trabeculae, which should be confirmed by further studying mouse embryonic hearts at the corresponding time point.

In conclusion, a mouse model of cardiomyocyte arhGEF18 gene cKO was successfully established. It was confirmed in model mice that knocking out the arhGEF18 gene can change the expression levels of the cytoskeleton and cell polar proteins in the myocardium. However, there is no obvious phenotype of LVNC in the constructed arhGEF18cKO mice at present. The model mice will be given external pathological stimuli or load tests, such as low temperature, running, and transverse aortic constriction (TAC) tests and a new arhGEF18cKO mouse model, such as arhGEF18^f/f^/Nkx2.5‐Cre^+^, will be constructed by changing the tool mice to further study the role and mechanism of arhGEF18 in relation to LVNC.

## AUTHOR CONTRIBUTIONS

Kun Wan performed the experiments, completed the overall data analysis and wrote the manuscript; Wenjing Yuan completed some of the data analysis; Tiewei Lv was responsible for the overall idea of the project and guiding the direction of the research, as well as revising the manuscript.

## CONFLICT OF INTEREST STATEMENT

The authors declare no conflict of interest.

## ETHICS STATEMENT

The experimental procedures described in this study were approved by the Experimental Ethics Committee of Children's Hospital of Chongqing Medical University (license number: CHCMU‐IACUC2021 1028001).

## Data Availability

The data that support the findings of this study are openly available in iReceptor at http://ireceptor.irmacs.sfu.ca/.
